# Useful Points of Value in a Country Practice

**Published:** 1898-09

**Authors:** S. S. Whitbeck

**Affiliations:** Decorah, Iowa


### USEFUL POINTS OF VALUE IN A COUNTRY PRACTICE.

By S. S. Whitbeck, D.V.M.,
DECORAH, IOWA.

I wish to call your attention to a few points in veterinary practice that may at least interest country practitioners, and hope through criticism to get the ideas of some of the best men in the country on the points mentioned.

The ideas here presented are written from a country practitioner's standpoint. We take it for granted that all city veterinarians have large, well-equipped, up-to-date hospitals. All country practitioners should have, at least, a small one of six to eight stalls, part single, part box-stalls, well padded all around for violent cases; force-pump or city water to flood floors with; small tank above for application of cold or hot water through a tube. The constant application of cold water to swollen shoulders and fistulous cases will nearly always remove the swelling and make the after-treatment much more simple and effective.

1 Read at the Thirty-fifth Annual Meeting of the United States Veterinary Medical Association, Omaha, Neb., September, 1898.

All recent bruises and strains are much benefited by a short course of cold applications. A fast horse was brought in one night that had just thrown out a good-sized curb by falling from an approach to the barn. He was so lame that he could hardly use the limb. Cold water was forced upon the enlargement through a small rubber tube over night, and the curb could hardly be seen in the morning, while the patient was resting the opposite leg. In intense fever and swelling after an operation, a cold stream forced upon the surrounding parts over night, and a teaspoonful of aconite tr., repeated in three to four hours, will generally arrest the dangerous symptoms in a few hours, and materially reduce the swelling. In fractures and severe contusions a hot stream is best, and is easily applied by means of a small oil-stove. Aconite may be used as above if there are symptoms of severe inflammatory action.

No one with any hospital practice should be without a stocks. They may be made in almost any design, well braced, and should always have a roller on each side to raise the patient from the floor when necessary. Any stocks can be used by one person, and will be found invaluable in detaining fistulous and poll-evil cases and for many of the minor operations. It saves time in looking up help, looks much better than casting every time you wish to touch a bad case, and is much safer to operator and patient alike than the twist alone. The writer has a stocks which cost seventeen dollars completed, and would prefer going without an operating-table rather than without the stocks. In it fractious cases can be safely dressed. Any part of the animal economy, from head to foot, can be made practically immovable, and a twist can be fastened to front standard by means of a staple and snap. While an operating-table furnishes a very nice method of detaining the patient for many operations, yet with the economical stocks on hand the table may be dispensed with. But one or the other is almost absolutely necessary in caring for most of our hospital cases.

I fear, however, that too many operators prefer the use of table and close confinement to the use of chloroform, and right here let me say that there is nothing in our practice that looks nicer or gives a better impression than properly chloroforming a patient for every operation that is at all severe. I believe that with a little more experimenting horses and cattle can be chloroformed almost instantaneously by the injection of chloroform into the jugular vein. I have tried this on five old horses, and several lumpy-jaw steers that were incurable, and have had no unfavorable results from it. The operation may be commenced at once, and if of any duration the anæsthesia may be prolonged by the application of ether and chloroform, half and half, to the nose. To date I have used from two to four drachms to one injection, and kept one patient under treatment two hours by inhalation of the mixture. All of the cases were subjects for the grave. Of the horses, two were killed after being kept under for an hour; the other three were up afterward for two hours, and injected again and killed. One of the steers is alive yet; two were kept for a day, and the others killed at once. But little chloroform is needed, and much time is saved. No one should use any of the anæsthetics without having amyl nitrate or nitroglycerin and hypodermic syringe at hand, although in my practice I have never had to use any restorative agent.

Many of the operations that were formerly very dangerous and painful are now performed with much less pain and little or no danger. In the operation on cryptorchids, for instance, we used to think it necessary to insert the hand and arm; but now nine out of ten cases can be operated upon by simply inserting one or two fore-fingers, and, working them in catch-fashion forward and out, the cord may be caught up and brought out, then the testicle. This method of performing the operation is very simple, and the danger is very slight, scarcely any more than in common colts. The patient should be thrown or placed with the head down hill, so the intestines will not be in the way; and the under rope should be brought back and up over the hip to the upper foot, so that the limbs cannot be extended and put the muscles in the pelvic regions to the strain. The nicest instrument for any castrating is the small-sized emasculator; bleeding is less liable to follow; the work can be done more quickly, and the instrument is more easily kept clean than the écraseur. True, in some cases, where the testicle is fastened internally, an écraseur is necessary, and may be passed into the abdominal cavity.

In deep-seated fistula cases, where the pus has burrowed between the shoulder-blades, the patient may be laid down in a soft bed, in as easy a position as possible, and kept down any length of time. One case of a very fractious disposition I laid down this way, and kept him there five weeks. He made a good recovery, all the matter escaping as soon as it formed, and the patient after a few days became as tame as a kitten, eating and drinking generally in the sitting posture, then lying down again. He was turned over three times a day by means of a pulley and rope.

Since listening to Prof. Reynolds's paper in Chicago I have tried both trephining and setoning. In trephining a little sac soon forms below and defeats us, but by passing a strong carbolized muslin-cloth from the bottom of the cavity down and out in front of the shoulder, a perfect dependent drainage is obtained, and most of the cases will do nicely.

In fracture of the limbs where splints or plaster-of-Paris bandages have to be applied, I very much prefer the keeping of the patient down, and find the owner generally prefers it to the slings. Of course, some of them will struggle for a time, but not as much as one would expect. A good many horses have more than horse-sense. With a good bed and oft turning there is but little danger of bedsores, and the patient will generally accommodate himself to the position very readily.

While there is plenty of room for advancement in veterinary surgery, there are likewise many things that we must learn yet in a medical line. One veterinarian, well up in his lines, writes: "We have no good treatment for bog-spavins, puffs, curbs, capped hocks, etc." I will admit that we know of nothing that works very quickly, but to the patient I suggest the following absorbent blister: Mercuric chloride, 1 oz.; gum camphor, 1 oz.; alcohol, 3 oz. Put mercury in solution in alcohol; then add turpentine to make a pint. Clip hair from the enlargement and wash clean. When dry rub in the above preparation with swab for three successive evenings. On the fourth morning wash clean and grease. Wash and grease every other day until the skin is healed, then repeat as before. Repeat thus five or six times, leaving the animal as quiet as possible in the meantime. In the soft enlargements bandages may be drawn tightly around the enlarged parts every evening after applying the blister, also during the interval between blisters, if desired. This does no harm in the treatment of curbs and capped hocks, rather hurrying the process of absorption. The bandages should be left on over night, while the patent arrangements for that purpose may be used several days at a time, the blistering with this making an ideal treatment, and nine times out of ten produces a satisfactory conclusion. After five or six series of blisters the patient should be turned in pasture or put at light work where there is no lameness remaining.

About two years ago I was called to see a sorrel mare, eight years of age, left hip shrunken very badly, bog-spavin extending all round the joint. The animal was so lame she could hardly step on her foot. I ordered the above blister alone, taking about two months to apply the six series of blisters, and then turned the patient out to pasture. In three months more the owner went out to get his mare, and did not know her at first, for the animal was as sound as any and round as an apple. But little of the enlargement was left, and this disappeared later on. The anmial has been used as a delivery horse since, and remains sound. Since then I have used this preparation on many curbs, capped hocks, puffs, and similar enlargements with excellent results.

The method of applying one blister after another as soon as the skin is healed until several have been applied, works very nicely on bone-spavins, ringbones, and splints. I have removed lameness frequently by this process, where firing has proved to be a failure. I paid over thirty dollars for a Paquelin cautery that I am now ready to make any young man a wedding present of. In fact, I believe that blistering, like any other form of treatment, must be kept up for some time if the best results are to be attained. The usual way of ordering one or two blisters is simply a starter.

In the treatment of black water the writer has found the lithium treatment to work very nicely in connection with bleeding. From three to six quarts may be withdrawn, according to the size of the patient, with decidedly beneficial results. Two to four drachms of the lithium may be given as a starter, and will generally be sufficient. Flax-gruel should be given every two hours at first, and thrice daily all through the attack. This serves as a soother to the kidneys, a laxative at first and a nutriment later. In all cases of azoturea I give ten to fifteen drops of 0.01 per cent. solution of nitroglycerin six to eight times intravenously, repeating the first two or three doses every hour, and every three hours through the attack. This lessens the tendency to spasmodic action of both voluntary and involuntary muscles, thus counteracting any tendency to sudden death; lessens the tendency to inflammation of the kidneys by promoting a flow of blood to the skin and extremities, regulates the heart's action, and, in my opinion, surpasses strychnine. As a nervo-muscular stimulant further on.

Nitroglycerin also makes an elegant stimulant in parturient apoplexy; given as above and in connection with other treatment, works equally as well in influenzas. In fact, any place where a quick nervo-vascular stimulant is needed, nitroglycerin is worthy of a trial.

In torpid conditions of the intestinal tract in horse or cow, in hoven, distention of the rumen with food, or indigestion of the third stomach, no better stimulant can be found than liquor ammonia acetatis in good-sized doses, having a powerful action on the entire glandular system. Most of my cases of impaction of the rumen are cured without any operation by the use of one pound of Epsom salts to begin with, and three-ounce doses of liquor ammonia acetatis, repeated every two hours until the bowels begin to move freely. I remember a bad case of this that had been treated by a country gentleman for one week ; then I was called in, and finding that plenty of cathartic had been given, I ordered four-ounce doses of this liquor every two hours until the bowels began to move freely, then every four hours. The cow was so weak at the commencement of my treatment that she could scarcely stand ; but by the next morning, she was much better, and made a nice recovery. Have tried it in poor digestion and constipation in human practice, with very satisfactory results ; two to four teaspoonfuls an hour before meals, often getting quite a laxative effect from its action alone. Indeed, the writer of this paper was bothered with dyspepsia, constipation, and (judging from my feelings at the time) nearly all the diseases the digestive tract is heir to. After trying most of the doctors' and all the remedies resorted to in such cases, I happened one sad day to think of the old cow and her marvellous recovery, and decided to try the treatment that had saved her, feeling that our troubles were somewhat mutual ; and to its action, no doubt, I owe the privilege and pleasure of being present with you to-day.

